# Plasmin Activation of Glial Cells through Protease-Activated Receptor 1

**DOI:** 10.1155/2013/314709

**Published:** 2013-01-28

**Authors:** André R. Greenidge, Kiana R. Hall, Ian R. Hambleton, Richelle Thomas, Dougald M. Monroe, R. Clive Landis

**Affiliations:** ^1^Edmund Cohen Laboratory For Vascular Research, Chronic Disease Research Centre, University of the West Indies, Cave Hill, Barbados; ^2^Division of Hematology/Oncology, School of Medicine, University of North Carolina, Chapel Hill, NC, USA

## Abstract

The objective of this study was to determine whether plasmin could induce morphological changes in human glial cells via PAR1. Human glioblastoma A172 cells were cultured in the presence of plasmin or the PAR1 specific activating hexapeptide, SFLLRN. Cells were monitored by flow cytometry to detect proteolytic activation of PAR1 receptor. Morphological changes were recorded by photomicroscopy and apoptosis was measured by annexinV staining. Plasmin cleaved the PAR1 receptor on glial cells at 5 minutes (*P* = 0.02). After 30 minutes, cellular processes had begun to retract from the basal substratum and by 4 hours glial cells had become detached. Similar results were obtained by generating plasmin de novo from plasminogen. Morphological transformation was blocked by plasmin inhibitors aprotinin or epsilon-aminocaproic acid (*P* = 0.03). Cell viability was unimpaired during early morphological changes, but by 24 hours following plasmin treatment 22% of glial cells were apoptotic. PAR1 activating peptide SFLLRN (but not inactive isomer FSLLRN) promoted analogous glial cell detachment (*P* = 0.03), proving the role for PAR1 in this process. This study has identified a plasmin/PAR1 axis of glial cell activation, linked to changes in glial cell morophology. This adds to our understanding of pathophysiological disease mechanisms of plasmin and the plasminogen system in neuroinjury.

## 1. Introduction

Plasmin is a serine protease best known for its thrombolytic properties in the coagulation system. However, it can also act on cells that bear receptors belonging to the protease-activated receptor (PAR) family to cause secretion of inflammatory cytokines, oxidative radicals, matrix metalloproteinases, proliferation, cell migration, and platelet aggregation [1–6]. PARs are widely expressed in the central nervous system [7].

Plasmin is generated from plasminogen, by proteolytic cleavage with either tissue-type plasminogen activator (tPA), urinary plasminogen activator (uPA), or bacterial streptokinase. It catalyzes the breakdown of fibrin into D-dimers, hence acting as a brake on coagulation. Antifibrinolytics are in clinical use to limit bleeding in cardiac surgery and intracranial bleeding in traumatic brain injury [8, 9]. Antifibrinolytics fall into two categories: lysine analogues that prevent plasmin generation from plasminogen, (e.g., *ε*-aminocaproic acid), or active site inhibitors (e.g., serine protease inhibitor aprotinin [10]).

A pathophysiological role has been recognized for the plasminogen-activating system in exacerbating intracranial bleeding, excitotoxicity and cell death in neurons or white matter, ischemia reperfusion injury, and increased permeability of the blood-brain barrier (BBB) [11–17]. Alterations in BBB permeability were accompanied by profound changes in cell shape to astroglial cell lines or primary glial cells, via an incompletely understood receptor mechanism [15]. This phenomenon has been confirmed in cardiac surgery patients with clinical evidence for glial cell injury [18]. 

PAR receptors share a common activation mechanism, whereby proteolytic cleavage unmasks a hexapeptide ligand sequence in the exodomain of the receptor, which can then dock intramolecularly and transmit G-protein-coupled signals into the cell [19]. PAR1 is activated by any serine protease that cuts specifically at arginine^41^-serine^42^ of the receptor generating the hexapeptide serine^42^-phenylalanine-leucine-leucine-arginine-asparagine^47^ (SFLLRN) at the new N-terminus. PAR1 activating proteases include thrombin, plasmin, and trypsin [20–22]. 

Although plasmin and PAR1 have independently been implicated in pathways of cerebral injury, a plasmin/PAR1 axis remains to be identified in cells of the central nervous system. In mice, genetic deletion of the PAR1 homolog or deletion of tPA confers neuroprotection in a model of transient cerebral ischemia [16, 22, 23]. Although plasmin has not been studied in this context, other PAR1 agonists trigger excitotoxicity, cell invasion, and apoptosis in neurons, astrocytes, and microglia [13, 16, 22, 24–28]. Hence, we investigated the ability of plasmin to activate central nervous system glial cells via PAR1 in an effort to identify key cellular events and highlight potential therapeutic targets.

## 2. Methods

### 2.1. Materials

Plasmin, plasminogen, streptokinase, aprotinin, and *ε*-aminocaproic acid were purchased from Sigma-Aldrich (St. Louis, MO). Phycoerythrin (PE) conjugated antibodies WEDE15(PE) and SPAN12(PE) were purchased from Beckman-Coulter (Caguas, PR). Phycoerythrin conjugated control antibody of the same isotype (IgG1-PE) was also purchased from Beckman-Coulter. PAR1 receptor activating hexapeptide (SFLLRN) and inactive scramble peptide (FSLLRN) were generated in house (University of North Carolina, Chapel Hill, NC) using an Applied Biosystems 432A Peptide Synthesizer (Life Technologies Corporation, Foster City, CA) and purified by reverse phase HPLC.

### 2.2. Cell Culture

The human glioma cell line A172 was purchased from the American Type Culture Collection (ATCC; Manassas, VA). Cells were maintained in Dulbecco's Modified Eagle's Medium (DMEM) enriched with 10% fetal calf serum, 2 mmoles/L L-glutamine, 100 U/mL penicillin, and 100 *μ*g/mL streptomycin (all Sigma-Aldrich) at 37°C in a humidified environment with 5% CO_2_. At confluence cells were detached using cell dissociation medium (Sigma Aldrich), a trypsin-free detachment step that avoids possible PAR1 activation by trypsin [20, 29]. For plasmin activation, cells were cultured to confluence in Corning Costar 24-well tissue culture plates (Sigma-Aldrich). Purified plasmin (Sigma-Aldrich) was used at 5 U/mL. Plasmin was also generated in situ from plasminogen and streptokinase (Sigma-Aldrich), monitoring plasmin generation spectrophotometrically with Spectrozyme colour reagent (American Diagnostica, Stanford, CT) at 405 nm using a Multimode Detector (Dynex Technologies, Chantilly, VA). Where indicated, aprotinin was used at 200 KIU/mL and *ε*-aminocaproic acid at 10 mmoles/L. SFLLRN or FSLLRN peptides were used at a concentration of 25 *μ*moles/L.

### 2.3. PAR1 Expression and Cleavage

PAR1 receptor expression and cleavage was carried out by flow cytometry as previously described [29]. Briefly, total receptor expression was assessed using the pan-reactive anti-PAR1 antibody WEDE15, whereas receptor activation due to plasmin cleavage was monitored using antibody SPAN12. SPAN12 detects only the intact (i.e., unactivated) PAR1 receptor; therefore, loss of SPAN12 staining provides a linear measure of receptor activation [30]. Staining was carried out on freshly passaged cells in suspension following stimulation with plasmin for 5 minutes at 37°C. Staining with WEDE15, SPAN12, or class-matched (IgG_1_) control antibody was carried out at 20 *μ*g/mL for 15 minutes on ice, followed by three washes in ice-cold PBS. Flow cytometric analysis was performed using an EPICS XL flow cytometer (Beckman Coulter). WEDE-15 or SPAN12 staining was expressed in units of relative fluorescent intensity (RFI), a ratio of the mean fluorescent staining intensity obtained with detection antibody versus the staining intensity of a class-matched control antibody (i.e., an RFI of 1.00 is equivalent to no detectable expression). Results were expressed as median ± IQR (RFI units).

### 2.4. Morphological Changes

Cells were observed and photographed at 0 min, 30 min, 4 h, and 24 h using a Leica DM IL inverted microscope (Leica Microsystems, Wetzlar, Germany) at ×40 or ×100 magnification. Morphological changes were scored on a 6-point ordinal scale according to the scheme: 1 = fully confluent lawn of cells; 2 = cellular pseudopodia have started to retract from one area; 3 = cell processes has started to retract from multiple areas; 4 = cell lawns show visible detachment from well substrata and flapping; 5 = cells have completely detached from well substrata to form a floating island; 6 = floating island of cells with shrivelled appearance.

### 2.5. Apoptosis

Apoptosis of A172 cells was monitored flow cytometrically by staining with Annexin V FITC Apoptosis Detection Kit according to the manufacturer's instructions (Sigma-Aldrich). The percentage of apoptosis was calculated by determining the area under the histogram greater than background staining in resting cells. Serum starvation was used as a positive control for apoptosis.

### 2.6. Statistics

Relative flourescent intensity and the morphological change scale were summarised using robust measures: median and interquartile range. Group differences were plotted using boxplots and assessed formally using the Wilcoxon-Mann-Whitney test for two-group comparisons. Statistical analyses used exact algorithms performed in Stata 10 (Stata Corp., College Station, TX).

## 3. Results

### 3.1. PAR1 Receptor Cleavage Induced by Plasmin

**Figure 1:**
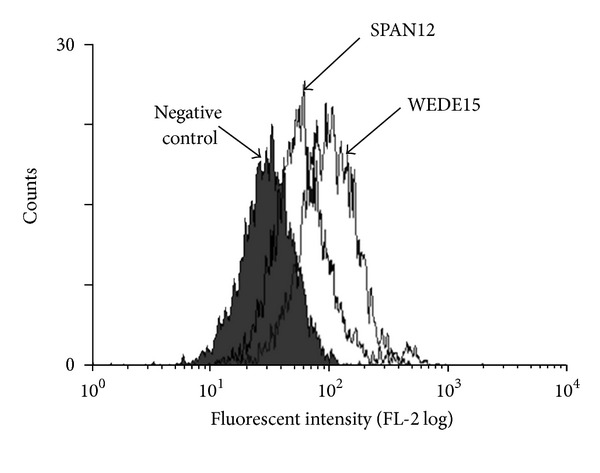
Expression of PAR1 epitopes. Flow cytometric histogram depicting expression of WEDE15 (a pan-receptor antibody) and SPAN12 (an activation-dependent antibody) on human A172 glioma cells. The filled histogram represents background staining with control antibody of the same isotype (IgG_1_).

**Figure 2:**
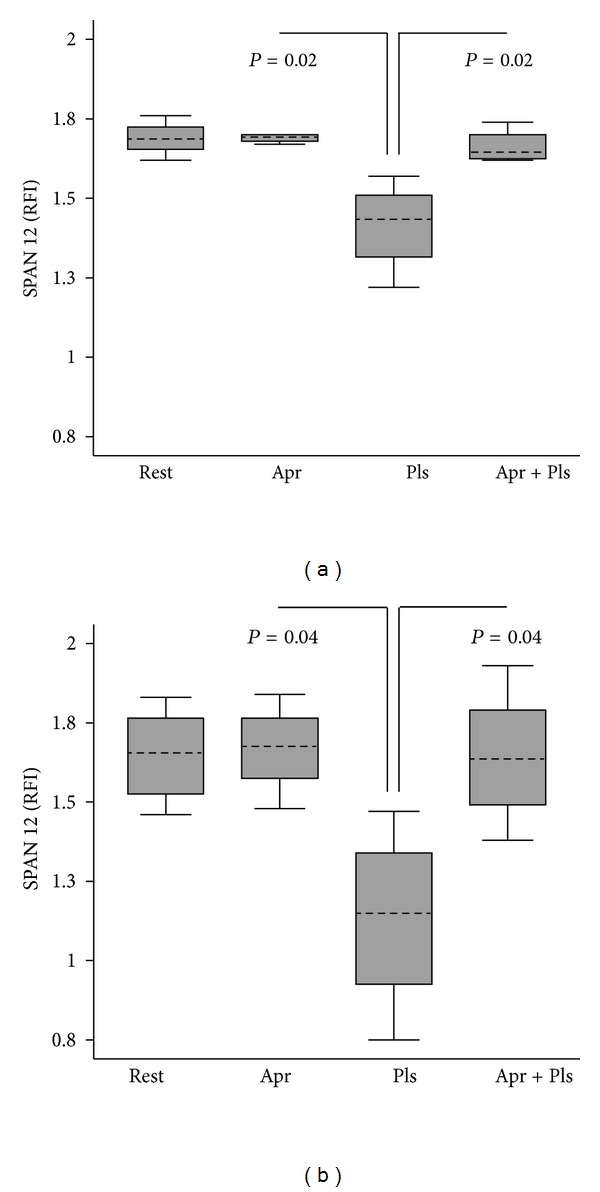
Effect of plasmin on PAR1 receptor activation. Proteolytic activation of PAR1 at 5 minutes was monitored flow cytometrically using antibody SPAN12 to detect intact (i.e., unactivated) receptor. Results were expressed in units of relative fluorescent intensity (RFI), calculated by dividing the mean fluorescent staining intensity obtained with SPAN12 antibody by the staining intensity obtained with a class matched (IgG_1_) control antibody. Results were expressed as the median ± interquartile range (IQR) from *n* = 4 experiments. (a) Preformed plasmin (5 U/mL). (b) Plasmin generated *in situ* from plasminogen. Rest = resting; Pls = plasmin; Apr = aprotinin 200 KIU/mL.

Initial experiments examined whether PAR1 was expressed on resting A172 glioma cells. A representative flow cytometric histogram illustrates expression of PAR1 detected with a pan-receptor antibody WEDE15 (Figure 1). WEDE15 staining from pooled samples (*n* = 8) demonstrated expression at an intensity of 2.31 ± 0.33 (median ± interquartile range (IQR)) relative fluorescent intensity units (RFI units) at the cell surface. Proteolytic activation of PAR1 due to plasmin was monitored using a different antibody, SPAN12, specific to only the intact (i.e., unactivated) receptor (Figures 1 and 2). This antibody was expressed at a fluorescent intensity of 1.69 ± 0.05 RFI units (median ± IQR.) on resting cells (*n* = 8). Activation with a purified plasmin preparation caused a statistically significant loss of SPAN12 expression at 5 minutes (1.69 ± 0.07 resting cells versus 1.43 ± 0.20 plasmin activated; *P* = 0.02; Figure 2(a)). To demonstrate whether this was due to proteolytic cleavage of the receptor by plasmin, experiments were repeated in the presence of the serine protease inhibitor aprotinin: this showed complete restoration of SPAN12 expression (1.65 ± 0.08; *P* = 0.02 versus plasmin alone). Analogous results were obtained when plasmin was generated *in situ* from plasminogen (Figure 2(b)).

### 3.2. Morphological Changes Induced by Plasmin

**Figure 3:**
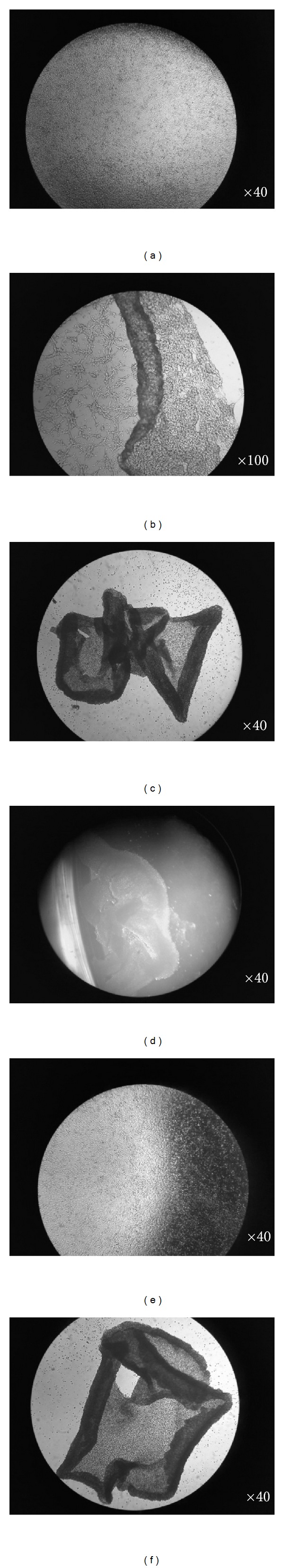
Effect of plasmin on cell morphology. (a) Time 0: confluent lawn of resting A172 glial cells. (b) 30 minutes after addition of plasmin: cell processes have started retracting from basal substrata, and flaps of detached cells were observed, although cell-to-cell contacts were maintained. (c) 4 hours: A172 cells fully detached into a floating island. (d) 24 hours: shrivelled floating cell mass. (e) 24 hours: aprotinin (200 KIU/mL) preserved the confluent monolayer of A172 cells in the face of plasmin activation. (f) 24 hours: a PAR1 agonist peptide SFLLRN (25 *μ*moles/L) triggered analogous glial cell detachment as observed for plasmin in (d). Control peptide FSLLRN did not trigger any morphological changes (not shown). The magnification is indicated at the bottom right of each panel. Representative photomicrographs from *n* = 3–5 experiments are shown.

**Figure 4:**
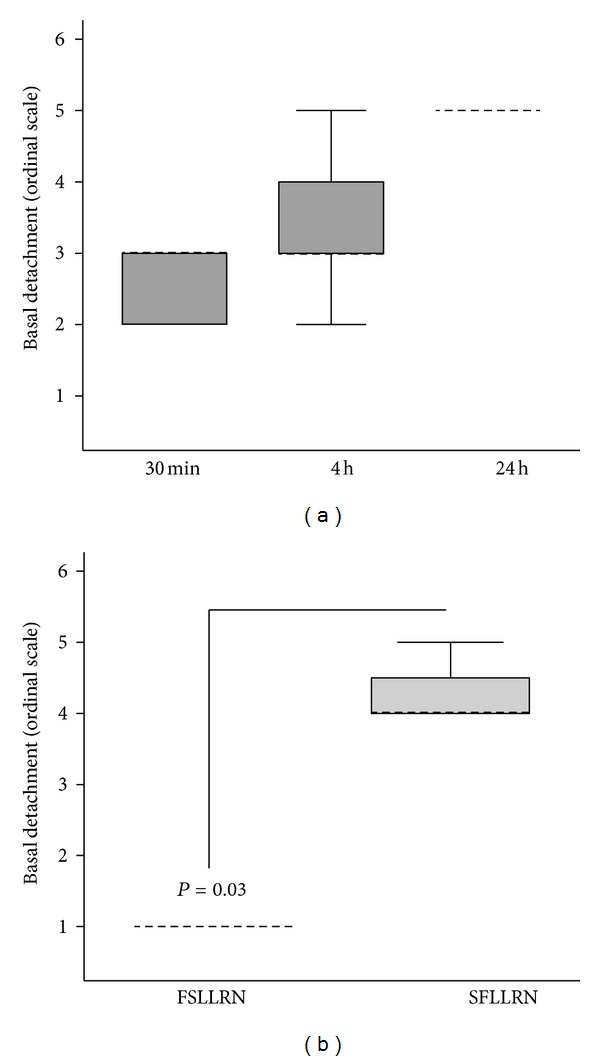
Effect of plasmin or PAR1 agonist peptide on glial cell detachment. (a) A172 cells were stimulated with plasmin (5 U/mL) for the length of time indicated, and cell detachment was quantified as defined in the Materials and Methods. (b) Effect of PAR1 specific activating peptide SFLLRN or inactive control peptide FSLLRN (both 25 *μ*moles/L) on cell detachment at 24 hours. Results were expressed as the median ± interquartile range (IQR) from *n* = 4 experiments.

**Figure 5:**
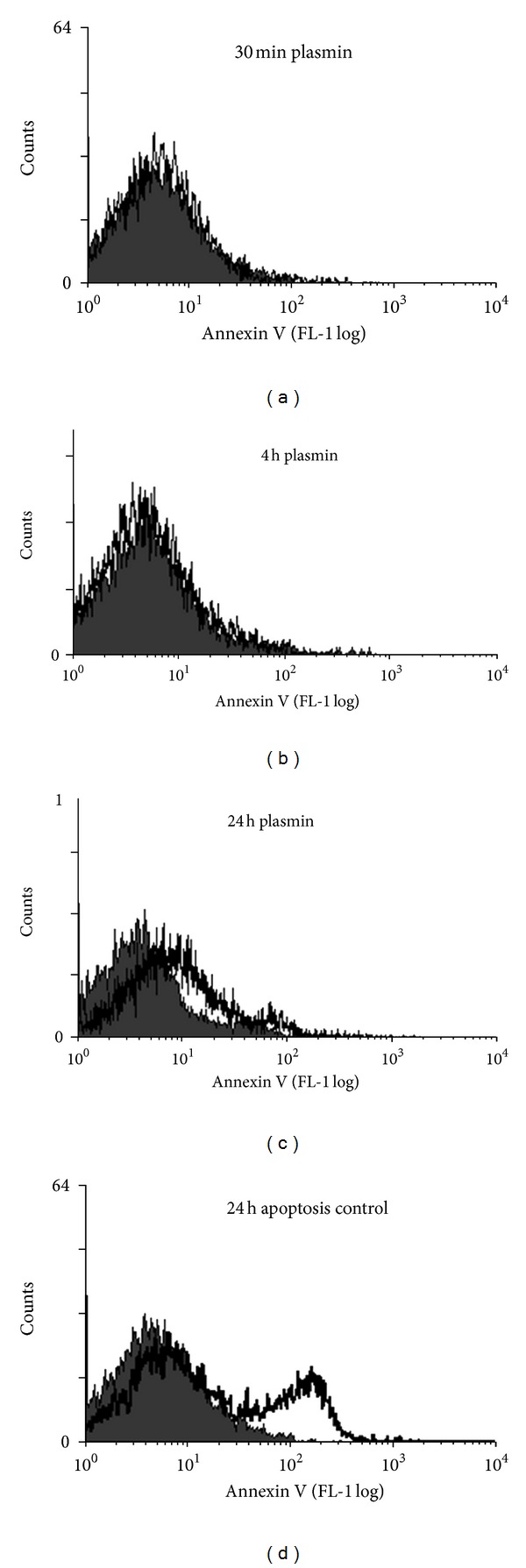
Effect of plasmin on glial cell apoptosis. A172 cells were stimulated with plasmin (5 U/mL) for the length of time indicated and monitored flow cytometrically for apoptosis (programmed cell death) by staining with Annexin V. Representative flow cytometric histograms depict the effect of plasmin (open histogram) versus resting cells (filled histogram) on Annexin V expression. Serum starvation was used as a positive control for apoptosis.

A timecourse of photomicrographs taken at 30 minutes, 4 hours and 24 hours after plasmin activation illustrates remarkable morphological transformation of A172 glioma cells (Figures 3 and 4(a)). At 30 minutes cell processes could be observed retracting from the basal substratum, and flap formation was seen at the edges of cell lawns (Figure 3(b)). By 4 hours the cell lawn had completely detached from the well into a floating island, apparently preserving cell-to-cell contacts (Figure 3(c)). Video microscopy showing cell detachment in real time illustrated the release of individual tethers from the end of cell processes (http://youtube/FkSUdWKfoxE). The morphological changes were abrogated by 200 KIU/mL aprotinin, indicating they were dependent on serine proteolytic activity of plasmin (Figure 3(e)). To demonstrate a specific role for PAR1 in this process, a PAR1 specific activating peptide SFLLRN (25 *μ*moles/L) was examined. This revealed exactly analagous morphological changes triggered through SFLLRN, but not through an inert scramble peptide FSLLRN even when used at high concentrations up to 250 *μ*moles/L (Figures 3(f) and 4(b)). Morphological changes up to 4 hours following plasmin treatment were not accompanied by any detectable apoptosis as assessed with Annexin V staining by flow cytometry (Figure 5). However, at 24 hours the floating mass of glial cells had a shrivelled appearance (Figure 3(d)) and flow cytometric analysis indicated 22% of cells were apoptotic (Figure 5). 

### 3.3. Effect of Antifibrinolytics

**Figure 6:**
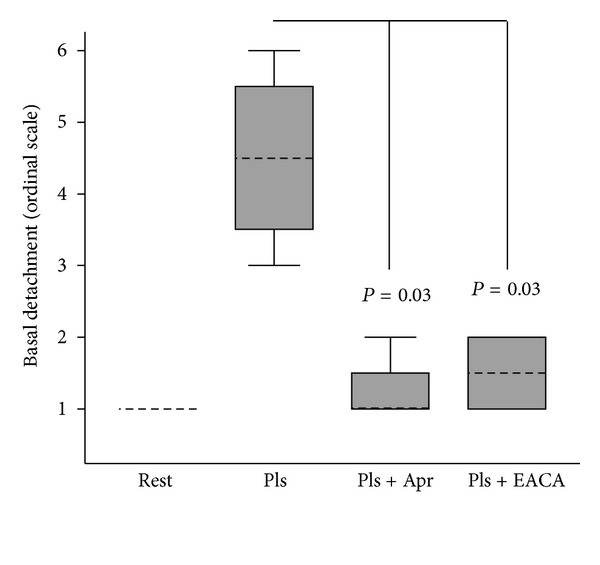
Effect of antifibrinolytics on plasmin induced glial cell detachment. A172 cells were stimulated with plasmin generated *in situ* from plasminogen (5 U/mL) and streptokinase (50 KU/mL) in the presence of aprotinin or *ε*-aminocaproic acid. Cell detachment was expressed in arbitrary units defined in Section 2. Results were expressed as the median ± interquartile range (IQR) from *n* = 4 experiments. Rest = Resting; Pls = plasmin; Apr = aprotinin (200 KIU/mL); EACA = *ε*-aminocaproic acid (10 mmoles/L).

The effect of antifibrinolytics on glial cell morphology was determined in cultures in which plasmin was generated *in situ* from plasminogen. The antiplasmin(ogen) agents aprotinin (200 KIU/mL) and *ε*-aminocaproic acid (10 mmoles/L), used at concentrations equivalent to their clinical usage, statistically significantly inhibited glial cell detachment due to plasmin (each *P* = 0.03; Figure 6). Biochemical assays for plasmin activity in cell cultures confirmed that each of the antifibrinolytics had abrogated plasmin activity at the concentrations employed (data not shown). 

## 4. Discussion

The present study has proven the principle that plasmin can activate human glial cells via proteolytic cleavage of PAR1. The type of cell-lawn detachment observed was similar to that previously described in A172 cells treated with an integrin antagonist, that also caused separation from basal substratum and accumulation of cells into floating spheroids [31]. Although immediate cytotoxicity due to plasmin was ruled out in apoptosis assays, gradual expression of annexin V occurred in our culture system after 24 hours following plasmin activation. The fact that SFLLRN (but not the inactive scramble peptide FSLLRN) could recapitulate the morphological changes in A172 cells proved the role of PAR1 in this process. 

These findings add to a growing literature that morphological transformation of astroglial cells can take place in a pathway involving the plasminogen activating system and components of the focal cell adhesion machinery [15, 31, 32]. An important recent study showed that tPA was capable of eliciting morphological changes and increased BBB permeability in mouse astroglial cells, through alterations to the Rho kinase (ROCK) pathway regulating focal adhesion contacts with extracellular matrix proteins [15]. A two-receptor mechanism was postulated in that study, one of which remained unknown. The unknown receptor was a receptor for plasminogen, as shown by antagonism with the antifibrinolytic agents aprotinin or tranexamic acid. The plasmin/PAR1 axis described by us was similarly antagonized by aprotinin or a lysine analog and may therefore present a plausible alternative receptor pathway. 

Most studies investigating the effect of serine proteases on neuronal PAR1 have focused on thrombin, which can either be neuroprotective or, conversely, induce apoptosis in neurons, depending on concentration and length of exposure to thrombin [7, 24, 25, 28, 33]. Initial studies using our culture system indicated that alpha-thrombin did not activate PAR1 on A172 glial cells within a physiological concentration range, but that plasmin activated PAR1 efficiently [34]. The plasmin/PAR1 axis of glial activation identified here may synergise to cause neurodegeneration with pathways of excitotoxic potentiation of N-methyl D-aspartate responses in neurons, also mediated via PAR1 activation with plasmin or thrombin [27, 35]. 

Cell lawn detachment may represent a manifestation of natural morphological processes, such as cell migration or astrocyte stellation. Reversal of the stellate phenotype by thrombin or changes in astrocyte morphology described for tPA may utilize the same cellular pathway, since both are regulated by ROCK [15, 36]. Local restructuring of focal adhesion contacts and cell detachment may emulate important physiological functions linked to cell migration, wound healing, proliferation, glial scarring, and neurite formation [17, 37–40]. Video microscopy of the cell detachment process appeared to indicate the release of individual pseudopod tethers, similar to what had been described for release or thinning of astrocyte cell processes induced by thrombin agonist [38, 41]. 

The morphological changes described here may be most relevant in the setting of neurotrauma or neuroinjury secondary to BBB breakdown, when zymogens such as plasmin that are normally confined to the systemic circulation can enter the brain [42]. A common surgical insult that compromises BBB function is cardiopulmonary bypass: this is linked to changes in glial cell morphology, neurocognitive deficits and stroke [18, 43–46]. The demonstration that glial cells are activated via plasmin/PAR1 expands our understanding of neurodegenerative mechanisms and focuses attention on PAR1 as a possible drug target for neuroprotection. While PAR1 deficient mice are protected in various models of ischemia, it is simplistic to consider PAR1 antagonism as a treatment option to block astroglial pathways of neuroinjury, since a clinical trial with the oral PAR1 antagonist vorapaxar for treatment of acute coronary syndromes was associated with significantly higher rates of intracerebral hemorrhage [22, 47, 48]. A desirable outcome would be to combine thrombolytic properties without hemorrhage or compromise of BBB permeability; however, this outcome remains elusive. However, plasmin inhibitors are neuroprotective, and the plasmin/PAR1 axis identified here may be explored further as a therapeutic target [17, 49–51].

There are some limitations to this study. The cell culture system may fail adequately to model the homeostatic environment of the brain, which is endowed not only with serine proteases and their zymogens but also naturally occurring serine protease inhibitors (serpins) [52, 53]. Furthermore, the *in vitro *cell culture model may not faithfully replicate *in vivo* pathways of glial injury caused by plasmin nor the three-dimensional cell-cell and cell-substrate interaction that arises from the precise cellular organization that exists within the brain. However, a reductionist cell culture approach as employed here is well suited to prove unequivocally that glial cells can be activated through proteolytic cleavage of PAR1 due to plasmin. 

In conclusion, we have identified a PAR1 axis of glial cell activation triggered by plasmin or the plasminogen system. This may be especially relevant under conditions of BBB breakdown or intracranial hemorrhage when serine proteases gain access to the neurovascular unit.
